# Experience-dependent changes in affective valence of taste in male mice

**DOI:** 10.1186/s13041-023-01017-x

**Published:** 2023-03-11

**Authors:** Shun Hamada, Kaori Mikami, Shuhei Ueda, Masashi Nagase, Takashi Nagashima, Mikiyasu Yamamoto, Haruhiko Bito, Sayaka Takemoto-Kimura, Toshihisa Ohtsuka, Ayako M. Watabe

**Affiliations:** 1 Department of Biochemistry, Faculty of Medicine, University of Yamanashi, Yamanashi, 409-3898 Japan; 2 Institute of Clinical Medicine and Research, Research Center for Medical Sciences, The Jikei University School of Medicine, 163-1 Kashiwashita, Kashiwa, Chiba 277-8567 Japan; 3 Department of Neuroscience I, Research Institute of Environmental Medicine, Nagoya University, Nagoya, 464-8601 Japan; 4 Department of Molecular/Cellular Neuroscience, Nagoya University Graduate School of Medicine, Nagoya, 466-8550 Japan; 5 Department of Neurochemistry, Graduate School of Medicine, The University of Tokyo, Tokyo, 113-0033 Japan

**Keywords:** Gustatory circuit, Umami, Bitter, Amygdala, Taste preference, Plasticity, Calcium imaging

## Abstract

Taste plays an essential role in the evaluation of food quality by detecting potential harm and benefit in what animals are about to eat and drink. While the affective valence of taste signals is supposed to be innately determined, taste preference can also be drastically modified by previous taste experiences of the animals. However, how the experience-dependent taste preference is developed and the neuronal mechanisms involved in this process are poorly understood. Here, we investigate the effects of prolonged exposure to umami and bitter tastants on taste preference using two-bottle tests in male mice. Prolonged umami exposure significantly enhanced umami preference with no changes in bitter preference, while prolonged bitter exposure significantly decreased bitter avoidance with no changes in umami preference. Because the central amygdala (CeA) is postulated as a critical node for the valence processing of sensory information including taste, we examined the responses of cells in the CeA to sweet, umami, and bitter tastants using in vivo calcium imaging. Interestingly, both protein kinase C delta (*Prkcd*)*-*positive and Somatostatin (*Sst*)-positive neurons in the CeA showed an umami response comparable to the bitter response, and no difference in cell type-specific activity patterns to different tastants was observed. Meanwhile, fluorescence in situ hybridization with *c-Fos* antisense probe revealed that a single umami experience significantly activates the CeA and several other gustatory-related nuclei, and especially CeA *Sst*-positive neurons were strongly activated. Intriguingly, after prolonged umami experience, umami tastant also significantly activates the CeA neurons, but the *Prkcd*-positive neurons instead of *Sst*-positive neurons were highly activated. These results suggest a relationship between amygdala activity and experience-dependent plasticity developed in taste preference and the involvement of the genetically defined neural populations in this process.

**Supplementary Information:**

The online version contains supplementary material available at 10.1186/s13041-023-01017-x.

## Introduction

The taste system is crucial for animals to detect potential benefits, e.g., nutrients, and potential harm, e.g., toxins, in what they are about to eat and drink [1, 2]. There are five basic taste qualities: sweet, sour, salty, bitter, and umami. The taste system has an emotional and motivational aspect, and affective taste, such as sweet and umami, drives approaching and appetitive behaviors, while aversive taste, such as bitter and sour, drives avoidance behaviors [3–5]. The attractive and aversive valence of taste signals is innately determined. For example, human newborn infants exhibit affective behaviors such as lip sucking, elevation of the corners of the mouth, and rhythmic tongue protrusions when exposed to sweet or umami solutions [6, 7]. They also show aversive responses such as nose wrinkling and grimacing when exposed to bitter or sour solutions. Rats and mice also exhibit affective behaviors such as rhythmic and lateral tongue protrusions when administered with sweet or umami solutions [7–9]. They also show aversive behaviors, such as gasping, chin rubbing, and handshaking, when exposed to quinine solution.

The attractive and aversive valence of taste can also be acquired so that experiences can modify the preference for certain tastants. For instance, many people may experience the development of an acquired taste for coffee, beer, or even quinine. Likewise, in rats and mice, exposure to sour and bitter substances before and after weaning leads to a significant preference for those substances in adulthood [10–12]. Thus, the unconditioned avoidance of sour and bitter can be modulated by early-life experiences. Compared with that of unfavored tastes, literature on the modification of hedonic valence of favored tastes is limited. It has been shown that rats exposed to overconsumption of sucrose during adolescence display reduced sweet consumption and hedonic perception in adulthood [13]. Furthermore, Ackroff et al. demonstrated that prior umami experience significantly enhances preference for umami solutions in mice [14]. These findings suggest that both attractive and aversive valence of taste signals can be subject to influence from previous experiences; however, the neuronal mechanisms underlying these observations are not well understood.

Taste signals first arise via taste receptor cells in the taste buds, which detect tastant chemicals and activate matching ganglion neurons. These signals are transmitted through gustatory nerves, including the chorda tympani and glossopharyngeal nerves, to the nucleus of the solitary tract (NTS), which relays information to the pontine parabrachial nucleus (PB) in rodents. These neurons then activate the ventral tegmental area (VTA), the ventral posteromedial nucleus of the thalamus, and the insular cortex (IC), which all project to the central amygdala (CeA) directly or indirectly [3, 15, 16]. Recent studies have demonstrated that taste information is processed by a labeled-line system, such that information about each taste quality has a discrete pathway from its taste receptors to the corresponding neuronal taste circuits [17, 18]. While some neurons in the central taste pathway are tuned to particular taste qualities, other neurons respond more broadly to multiple tastes [19]. Furthermore, some neurons change in their responsive profiles according to experience and time [20], which suggests some plasticity in the taste coding rule. Therefore, neural plasticity within the described taste circuitry can regulate experience-dependent changes in the affective and aversive valence of particular taste signals.

In the present study, we have addressed this issue by establishing a behavioral paradigm for experience-dependent plasticity in the taste preference for attractive (umami) and aversive (bitter) taste quality, and examined the neuronal correlates in mice using in vivo calcium imaging and fluorescence in situ hybridization in multiple brain regions.

## Materials and methods

### Animals

Adult male C57BL/6J mice (Japan SLC, Inc., Shizuoka, Japan) were group housed (3–4 mice per cage) on a 12 h light/12 h dark cycle and provided with food (CE-2, CLEA Japan, Inc., Tokyo, Japan) and water ad libitum, unless otherwise noted. Protein kinase C delta (*Prkcd*)-cre mice [Tg(Prkcd-glc-1/CFP,-cre)EH124Gsat; stock #011559-UCD] and somatostatin (*Sst*)-cre mice [Sst tm2.1(cre)Zjh/J; stock #013044] were obtained from the Mutant Mouse Resource & Research Center and the Jackson Laboratory, respectively, and were maintained heterozygous on a C57BL/6J background. Adult male mice over 3 months old were used for in vivo calcium imaging studies. All experimental protocols in this study that included the use of animals were approved by the Institutional Animal Care and Use Committee of The Jikei University (Kashiwa City, Japan) (Approval number 2018-072, 2019-010) and Nagoya University (approval number R210154). All experiments complied with the Guidelines for Proper Conduct of Animal Experiments by the Science Council of Japan (2006) and those recommended by the International Association for the Study of Pain. All efforts were made to reduce the number of animals used and the suffering of the animals.

### Prolonged taste exposure and two-bottle tests

Mice in the prolonged taste exposure groups had ad libitum access to food and one of the following taste solutions instead of water beginning at 4 weeks of age, immediately after weaning, for 3 weeks. The umami solution contained 100 mM monosodium l-glutamate (Sigma-Aldrich, Darmstadt, Germany) or 100 mM monopotassium l-glutamate (Sigma-Aldrich, Darmstadt, Germany) and 10 mM disodium inosine-5′-monophosphate (Sigma-Aldrich) mixture. The bitter solution contained 0.3 mM quinine hydrochloride (FUJIFILM Wako, Osaka, Japan). Mice that experienced prolonged taste exposure were subjected to a two-bottle test following 19–21 h of water deprivation. Mice were acclimated to the stainless steel sipper tubes in the two-bottle test chamber (Drinko-measurer; DM-G1, trapezoid-shaped test chamber with 55 mm upper side × 205 mm lower side × 135 mm depth × 200 mm height; TOP-3002WW, O’Hara & Co., Ltd, Tokyo, Japan) within the sound-attenuating box (660 mm width × 460 mm depth × 690 mm height; TOP-4011, O’Hara & Co., Ltd) for 15 min per day for 4 days. The two-bottle test was performed with the bottle positions switched for 2 days to avoid side preference. On test day 1 and day 2, an umami solution bottle and a water bottle were presented. On day 3 and day 4, a bitter solution bottle and a water bottle were presented. Access duration to each bottle was measured as nose poking time to the bottle and analyzed by Operant task Studio V2 (O’Hara & Co., Ltd). A preference ratio was calculated as the ratio of the taste solution (umami or bitter) intake to the total (taste solution and water) intake. An access ratio was determined as the ratio of the access duration in the taste bottle to the total access duration in two bottles. Mice in the Umami and Bitter groups that exhibited an intake of less than 0.1 g during the 15-min test were excluded from the data because of the inability to properly assess their preference. After the 15-min test, the mice were returned to the home cage and given ad libitum access to food and taste solution until the next test on the following day.

### Stereotaxic surgery for in vivo calcium imaging

Mice surgeries were performed as described previously with minor modifications [21, 22]. Each deeply anesthetized mouse was fixed in a stereotactic frame (Model 942; Kopf Instruments, CA, USA). For viral injection, skull surface was exposed, a glass capillary was inserted through a drilled small hole, and 500 nL of adeno-associated virus (AAV) solution (AAV1/2-CAG-DIO-GCaMP6f-WPRE; 3.5 × 10^13^ genomes/mL) [23] was loaded into the right CeA (AP − 1.35, ML + 2.95, DV − 4.60) according to the atlas [24]. More than a week after viral injection, a second surgery was performed to implant a customized 0.6-mm-diameter gradient index (GRIN) lens probe (Inscopix, CA, USA) on the right CeA (AP − 1.45, ML + 3.00, DV − 4.40) using a custom-made implanter. The implanted lens probe was fixed to the skull using UV-curable optical adhesive (NOA-81; Norland Products, NJ, USA). Exposed skull was coated with super-bond (C&B Kit; Sun Medical, Shiga, Japan), additionally covered with dental cement (REPAIRSIN; GC, Tokyo, Japan), and a stainless steel bar was attached for head fixation. GCaMP6f fluorescence was periodically evaluated using a miniature integrated microscope system (nVista HD 2.0; Inscopix), and when sufficient GCaMP6f expression was confirmed, the microscope baseplate was mounted using blue light curing resin (Flow-It ALC; Pentron, CT, USA). The sites of viral injection and lens probe implantation were confirmed histologically after imaging experiments (Additional file 3: Fig. S3).

### In vivo calcium imaging during taste stimulation

More than 5 days after baseplate mounting, the mice were ready for imaging. After several days of imaging studies for natural experiences, imaging experiments for taste stimuli-evoked responses were performed. A detailed procedure of the precedent imaging studies will be described elsewhere. A day before the experiment, the miniscope was attached to each mouse and each mouse was head-fixed on a running disc wheel for 30 min to acclimatize, and then 100 µL of water was given six times at 2-min intervals using an oral gavage ball tip needle. On the experimental day, mice were habituated for 30 min as on the previous day, and calcium imaging was performed during taste stimulation. Images were acquired using data acquisition software (ver. 2.0.4; Inscopix) at 20 frames per sec, 25% of LED power, and a gain of 3.5, and behavioral videos were recorded simultaneously by triggers from the miniscope system. Six minutes after the start of imaging, mice were given 100 µL of bitter (1 mM quinine), sweet (50 mM sucrose), and umami (100 mM monosodium glutamate and 10 mM disodium inosinate) taste solution alternately with neutral-taste water at 2-min intervals for three trials (Fig. 2A).

Acquired imaging data were down-sampled (1/2 spatial binning), preprocessed, motion corrected, cropped, and then additionally down-sampled (1/2 spatial and 1/2 temporal binning) using Mosaic Software (ver. 1.2.0; Inscopix). Processed images were loaded to Inscopix Data Processing Software (ver. 1.3.0; Inscopix), and then calcium transients of individual neurons were extracted with a constrained non-negative matrix factorization for microendoscopic data (CNMF-E) [25] with MATLAB (ver. R2018b; MathWorks, MA, USA). All extracted traces were manually checked, and traces from multiple cells or non-cellular signals were excluded. Fluorescent traces from each neuron were z-scored, and taste-activated neurons were defined by the following formula: (averaged z-score for 30-s after each taste stimulation at three trials − averaged z-score for 30-s after water given at nine trials) > 0.5.

### *Fos* counting by fluorescence in situ hybridization

The preparation of complementary RNA (cRNA) probes and fluorescence in situ hybridization (FISH) were performed as described previously [26], with some modifications. To construct *Fos,* nitric oxide synthase 1 (*Nos1*), *Prkcd*, *Sst*, tyrosine hydroxylase (*Th*), calcitonin gene-related peptide (*Calca*), and pituitary adenylate cyclase-activating polypeptide (*Adcyap1*) FISH probes, total RNA from the adult B6 mouse brain was reverse transcribed by Prime Script II RTase (Takara Bio Inc., Shiga, Japan), and the *Fos* (NM_010234.2, 1–1291 base), *Nos1* (NM_008712.1, 2898–3648 base), *Prkcd* (NM_011103.3, 238–2262 base), *Sst* (NM_009215.1, 7–550 base), *Calca* (NM_007587.2, 156–566 base), and *Adcyap1* (NM_009625, 1244–2103 base) sequences were amplified by polymerase chain reaction using PrimeSTAR MX DNA Polymerase (Takara Bio Inc.) with specific primer sets (Table 1). The resulting polymerase chain reaction fragments were subcloned into pBlueScript II KS (+) phagemids (Agilent, CA, USA). TH-inserted pBlueScript plasmid was kindly gifted from Prof. Watanabe (Hokkaido Univ.) [27]. Fluorescein isothiocyanate (FITC)- or Digoxigenin (DIG)-labeled cRNA probes were prepared using T3 or T7 RNA polymerase (Promega, WI, USA) with a FITC or DIG RNA labeling mix (Roche Diagnostics, Tokyo, Japan) at 37.0 °CC for 2 h.

**Table 1:** Cloning primers for FISH probes

Primer name	Sequence
*Fos*_F	gggctgcaggaattcCAGCGAGCAACTGAGAAGAC
*Fos*_R	cccctcgaggtcgacTCTGACTGCTCACAGGGCCA
*Nos1*_F	cgggctgcaggaattcGGCTAAGAAAGTCTTCAAGG
*Nos1*_R	ccccctcgaggtcgacACATGTCTGGAGAGGAGCTG
*Prkcd*_F	cgggctgcaggaattcATGGCACCCTTCCTGCGCATC
*Prkcd*_R	ccccctcgaggtcgacTTAAATGTCCAGGAATTGCTC
*Sst*_F	cgggctgcaggaattcTGAAGGAGACGCTACCGAAG
*Sst*_R	ccccctcgaggtcgacTGCAGGGTCAAGTTGAGCATC
*Calca*_F	tcccccgggctgcagATGGGCTTCCTGAAGTTCTC
*Calca*_R	cccctcgaggtcgacTGCCAAAATGGGATT
*Adcyap1*_F	accgcggtggcggccgcTGGGTGCACAAGGATTGAA
*Adcyap1*_R	ccccctcgaggtcgacGGCAAGGGTAGGAAGGAGGG

Lowercase letters indicate overlap sequence for cloning into pBluescriptII KS. Underlines indicate restriction enzyme sites. Uppercase letters indicate the recognition sequence for each gene

Six-week-old naïve and prolonged taste exposed mice were acclimated to test bottles for 3–4 days before the stimulation. Following 19–21 h of water deprivation, each mouse was placed in the stimulation cage (300 mm × 160 mm × 140 mm) with clean paper bedding without food and water under a dim light 1 h before taste stimulation as the adaptation experimental environment. Then, mice were presented with a bottle with stainless steel sipper tubes (Drinko-measurer; DM-G1, O’Hara & Co., Ltd.) containing either water or umami solution under free-moving condition. Thirty minutes after the first licking action, the animals were deeply anesthetized with isoflurane (5%) and sacrificed for in situ hybridization. Brains were removed, frozen rapidly by dry ice, and stored at − 80 °C. The frozen brains were sectioned coronally at a thickness of 20 μm on a cryostat (HM525 NX, Thermo Fisher Scientific, MA, USA) at the Bregma + 1.10 to + 0.60 (IC), − 1.22 to − 1.58 (CeA), − 3.08 to − 3.40 (VTA), − 5.02 to − 5.40 (PB), and − 6.84 to − 7.92 (NTS). Sections mounted onto glass slides were fixed with 4% paraformaldehyde and treated with the following acetylation and hybridization buffers. Acetylation buffer: 0.25% acetic anhydride and 0.1 M triethanolamine-HCl (pH 8.0); hybridization buffer: 50% formamide, 600 mM NaCl, 33 mM Tris–HCl (pH 8.0), 1 × Denhardt’s stock solution, 10% dextran sulfate, 1 mM EDTA, 0.1% *N*-Lauryl sarcosine sodium salt, and 200 μg/mL tRNA. Hybridization was performed for 12–16 h at 63.5 °C with FITC-labeled *Fos* and DIG-labeled cell type-specific marker cRNA probes in hybridization buffer. Subsequently, sections were washed at 61.0 °C with 5 × standard saline citrate (SSC) for 30 min, 50% formamide containing 4 × SSC for 15 min, 50% formamide containing 2 × SSC for 15 min, and 0.1 × SSC for 30 min three times. Additional washing steps were performed at room temperature using NTE buffer [0.5 M NaCl, 10 mM Tris–HCl (pH 8.0), and 5 mM EDTA] for 5 min, NTE buffer containing 20 mM iodoacetamide for 15 min, NTE buffer for 10 min, and TNT buffer [0.1 M Tris–HCl (pH 7.4) and 0.15 M NaCl] for 5 min (the latter was used as a washing buffer in subsequent processes). Samples were incubated with DIG blocking buffer [1% blocking reagent (Roche Diagnostics) and 10% normal sheep serum (Merck Millipore, MA, USA)] for 30 min, and incubated with 0.5% (wt/vol) TSA-blocking solution (Akoya Biosciences, MA, USA) for 30 min. The detection of the FITC-labeled probe was performed using a peroxidase-conjugated anti-FITC antibody (Roche Diagnostics), followed by processing with TSA plus the FITC System (Akoya Biosciences). After the inactivation of peroxidase by 1% hydroperoxide, detection of the DIG-labeled probe was performed using a peroxidase-conjugated anti-DIG antibody (Roche Diagnostics) with 4′,6-diamidino-2-phenylindole (DAPI) for 1 h, followed by processing with TSA plus the Cyanine 3 System (Akoya Biosciences). Fluorescent images were acquired using an FV1200 (Olympus, Tokyo, Japan) microscope equipped with a dry objective (UPlanSAPO 10X/0.40, Olympus) for analysis of FISH signal intensity.

For cell counting, imaging analysis was performed using expanded ImageJ version Fiji (NIH). ROI areas were determined by the marker expression pattern. Images were converted into the binary pattern using auto-threshold algorithms (“Triangle” for *Fos* and “Moments” after background subtraction for *Prkcd*, *Sst*, *Calca*, and *Adcyap1*) and particles more than 10 µm^2^ were analyzed. The irrelevance signals, such as non-match to the DAPI signal and two or three divided signals in one nucleus, were corrected manually. *Fos* and marker double-positive neurons were counted as cells with more than 4 pixels overlapped. Data were normalized by the ROI area or the number of marker-positive cells. In data tabulation, *Fos* FISH counts were analyzed for each slice, and the 4 slices with middle value for each individual were used for tabulation in order to reduce variation among individuals. The four slices with middle value were also adopted for analyses of the double-staining with *Fos* and cell-type marker genes in the CeA and PB.

### Quantification and statistical analysis

The intake and access duration were analyzed using paired *t*-test. The preference ratio and access ratio were analyzed by one-way ANOVA followed by Tukey’s post hoc test and one sample *t*-test. The *Fos* FISH cell counting data were analyzed by unpaired *t*-test.

## Results

### Chronic umami or bitter exposure induced increased preference for umami or decreased aversion to bitter

**Fig. 1:**
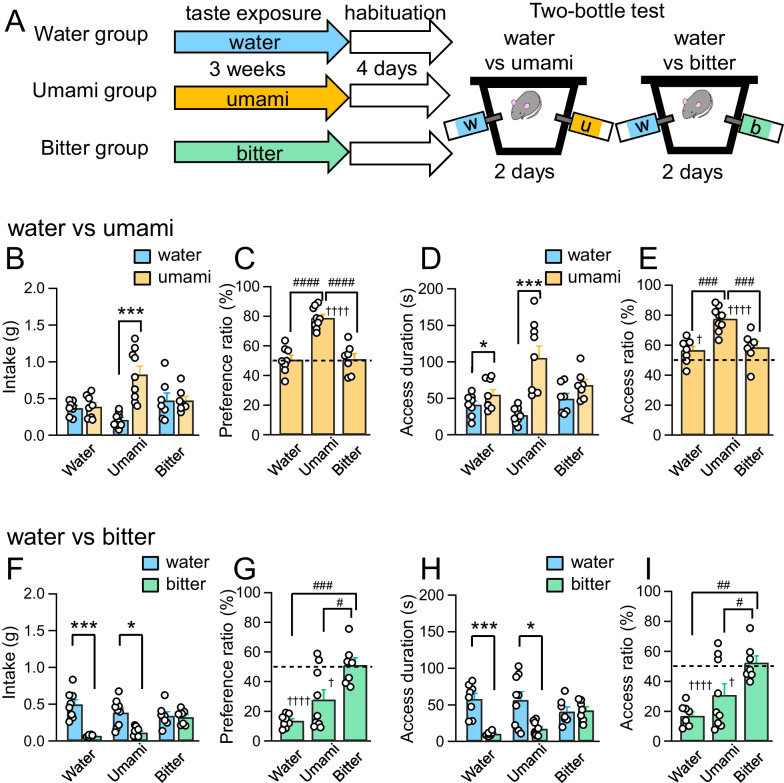
Preference for umami or bitter in the two-bottle test in prolonged taste exposure mice. **A** Experimental paradigm of prolonged taste exposure and two-bottle test. **B** Intake of water and umami during 15-min two-bottle test. **C** Preference ratios of umami. Preference ratios were calculated as the ratio of the umami intake to the total intake. **D** Access duration to water or umami bottle. **E** Access ratio of umami bottle. **F** Intake of water and bitter during 15-min two-bottle test. **G** Preference ratios of bitter. **H** Access duration to bitter bottle. **I** Access ratio of water or bitter bottle. Each circle represents results from one mouse. Data are represented as mean ± SEM. Water group, n = 8; Umami group, n = 9; Bitter group, n = 7. *p < 0.05, ***p < 0.001 (paired *t*-test); ^#^*p* < 0.05, ^##^*p* < 0.01, ^###^*p* < 0.001, ^####^*p* < 0.0001 (Tukey’s post hoc test); ^†^*p* < 0.05, ^††††^*p* < 0.0001 (one sample *t*-test)

To investigate the influence of prolonged experience of umami and bitter tastants on taste preference, mice were reared with ad libitum water (Water group), umami solution (Umami group), or bitter solution (Bitter group) for 3 weeks in the immediate post-weaning period (Fig. 1A), which did not affect body weight gain (Water group, 4.45 ± 0.32 g; Umami group, 3.96 ± 0.22 g; Bitter group, 4.10 ± 0.42 g). After prolonged taste exposure, we performed the two-bottle test between water and umami to assess umami preference. The Umami group exhibited a significant increase in intake of umami solution compared with water, whereas the Water and Bitter groups showed no difference in water and umami intake (Water group, *p* = 0.6588; Umami group, *p* = 0.0003; Bitter group, *p* = 1.0000; Fig. 1B). Total intake of both water and umami was comparable between the three groups (Water group, 0.76 ± 0.08 g; Umami group, 1.04 ± 0.13 g; Bitter group, 0.94 ± 0.15 g). We calculated the ratio of umami intake to total intake of water and umami as a preference ratio, so that a preference ratio higher than the 50% value indicated that umami was preferred over water. The preference ratio of umami in the Umami group was significantly higher than 50% (*p* < 0.0001; Fig. 1C). In addition, comparison of the preference ratios among the three groups revealed that the Umami group showed a significantly high preference ratio of umami (F_2,21_ = 30.09, *p* < 0.0001; Umami vs. Water group, *p* < 0.0001; Umami vs. Bitter group, *p* < 0.0001; Water vs. Bitter group, *p* = 0.9934; Fig. 1C), which indicated that prolonged exposure to umami increased its preference. We also analyzed access duration to the water and umami bottles to assess exploring behavior to each tastant. All groups showed significant increase in access duration to the umami bottle at several time points when analyzed every 5 min (Additional file 3: Fig. S1A–C). Therefore, potential neophobia to the unexperienced tastants, which may have been observed in the first 5 min, would have been canceled or at least negligible in our experimental condition. In total access duration during the whole test session, both the Water and Umami groups contacted the umami bottle longer duration than the water bottle (Water group, *p* = 0.0315; Umami group, *p* = 0.0008; Fig. 1D). The Bitter group showed a tendency of increased access duration to the umami bottle (*p* = 0.0676; Fig. 1D). The ratio of access duration to the umami bottle in the Water and Umami groups was significantly high compared with the chance rate (50%), and that of the Bitter group was slightly but not significantly higher than 50% (Water group, *p* = 0.0483; Umami group, *p* < 0.0001; Bitter group, *p* = 0.0676; Fig. 1E). These observations indicate that not only the Umami group but also the Water and Bitter groups showed interest in umami. The Umami group, however, had a significantly higher access ratio to the umami bottle compared with that of the other groups (F_2,21_ = 15.12, *p* < 0.0001; Umami vs. Water group, *p* = 0.0002; Umami vs. Bitter group, *p* = 0.0008; Water vs. Bitter group, *p* = 0.9063; Fig. 1E).

To further confirm that the experiments with umami (100 mM monosodium glutamate, MSG) reflects umami effects rather than sodium effects, we also investigated the influence of the prolonged experience of umami (100 mM monopotassium glutamate, MPG). Mice were reared with ad libitum water (Water group), MPG-based umami solution (MPG group) for 3 weeks in the immediate post-weaning period (Additional file 3: Fig. S2A), which did not affect body weight gain (Water group, 5.32 ± 0.36 g; MPG group, 5.67 ± 0.35 g). We found that MPG experience for 3 weeks enhanced umami intake similar to MSG experience (Water group, *p* = 0.0261; Umami group, *p* = 0.0047; Additional file 3: Fig. S2B, Fig. 1B). Although both the Water and MPG groups showed attraction to umami, the MPG group exhibited higher preference ratio (MPG vs. Water group, *p* = 0.1336; Additional file 3: Fig. S2C). In addition, the MPG group exhibited increased access duration and access ratio compared to the Water group (Additional file 3: Fig. S2D, E). These results regarding access duration are similar to those obtained using MSG. Taken together, these experiments support the idea that our experiments with MSG also reflect the effect of umami. These results using MSG and MPG suggest that prolonged umami exposure increased the preference for umami. The following experiments were conducted with MSG-based umami solution that induced remarkable changes in umami preference in behavioral experiments.

Next, we performed the two-bottle test with water and bitter solution to assess whether prolonged umami or bitter exposure affected bitter aversiveness. The Water and Umami groups consumed significantly less bitter solution than water (Water group, *p* = 0.0004; Umami group, *p* = 0.0165; Fig. 1F) and showed small preference ratios of bitter compared to 50% (Water group, *p* < 0.0001; Umami group, *p* = 0.0113; Fig. 1G). In contrast, the Bitter group consumed as much bitter solution as water (*p* = 0.7539; Fig. 1F) and exhibited bitter preference ratio around 50% (*p* = 0.8361; Fig. 1G), which was significantly high compared with that of the other groups (F_2,21_ = 12.37, *p* = 0.0003; Umami vs. Water groups, *p* = 0.1372; Umami vs. Bitter groups, *p* = 0.0128; Water vs. Bitter groups, *p* = 0.0002; Fig. 1G), indicating that the Bitter group showed no aversion to bitter. The total intake of water and bitter was comparable between the three groups (Water group, 0.56 ± 0.07 g; Umami group, 0.49 ± 0.05 g; Bitter group, 0.66 ± 0.08 g). Although access duration to the bitter bottle was significantly less than that to the water bottle in the Water and Umami groups, the Bitter group accessed the bitter and water bottles for almost the same duration (Water group, *p* = 0.0005; Umami group, *p* = 0.0198; Bitter group, *p* = 0.8296; Fig. 1H; Additional file 3: Fig. S1D–F). The access duration ratio to the bitter bottle in the Bitter group was significantly higher than that of the other groups (F_2,21_ = 9.17, *p* = 0.0014; Umami vs. Water group, *p* = 0.2014; Umami vs. Bitter group, *p* = 0.0370; Water vs. Bitter group, *p* = 0.0010; Fig. 1I). These results suggest that prolonged bitter exposure decreased aversion to bitter, which was innately aversive.

### The CeA is composed of neurons with heterogeneous response properties for various tastants

**Fig. 2:**
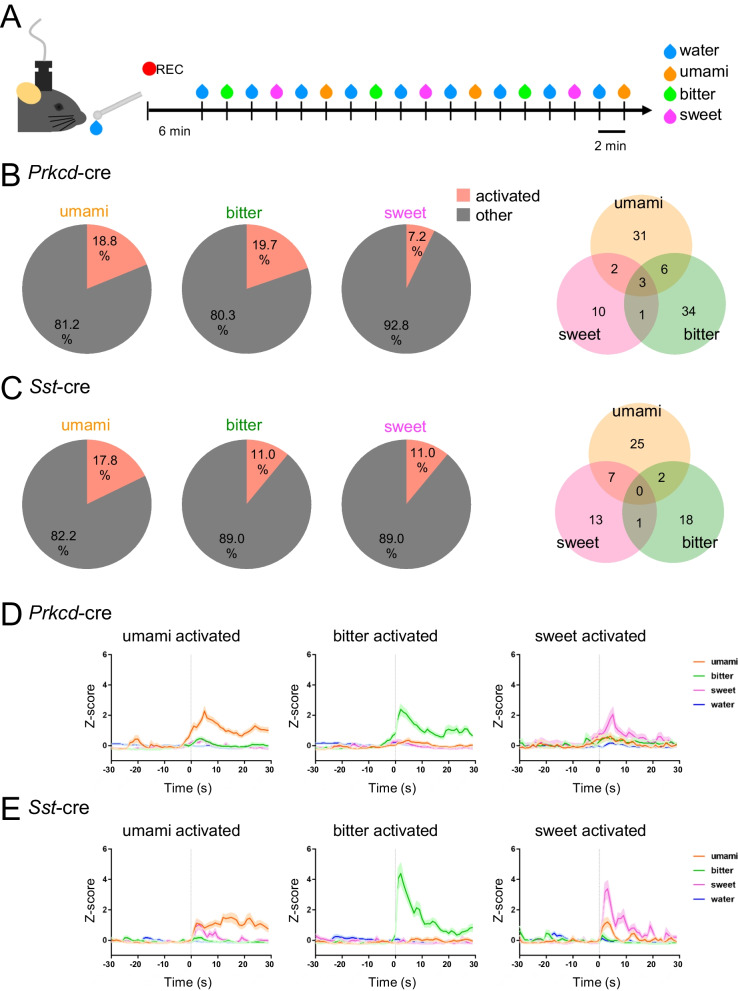
In vivo calcium imaging of central amygdala (CeA) neurons during taste stimulation. **A** Schematic of drinking experiment for calcium imaging of taste stimuli-evoked responses. **B**, **C** Pie charts showing the fraction of response cells for each taste in the total cell population (**B** 223 cells from four *Prkcd-*cre mice, **C** 191 cells from four *Sst-*cre mice). Venn diagrams showing the overlap of activated cells. **D**, **E** Average z-scored GCaMP6f signals of umami-activated (42 cells from *Prkcd-*cre mice and 34 cells from *Sst-*cre mice), bitter-activated (44 cells from *Prkcd-*cre mice and 21 cells from *Sst-*cre mice), and sweet-activated (16 cells from *Prkcd-*cre mice and 21 cells from *Sst-*cre mice) cells in response to umami (orange), bitter (green), sweet (magenta), and neutral (blue) tastant solution stimuli. Shading, ± s.e.m

These changes in taste preference/avoidance due to prolonged taste exposure were considered as an adaptation accompanied by neuroplasticity. We next sought to determine the areas of the brain that display neuronal activity associated with these behavioral changes. Recent studies have reported that the CeA plays a pivotal role in emotional behavioral selection [28]. In addition, the CeA receives direct input from multiple nuclei of the gustatory circuit such as the NTS, PB, and IC [5, 29, 30]. Especially, it has been reported that *Prkcd*-positive neurons in the CeA are a population that responds to aversive tastant [2]. Therefore, one intriguing possibility is that there are cell-type specific responses to the negative and positive taste qualities within the CeA, and neuronal activity changes occur in this circuit may lead to the modification of outcome behavior toward the tastant. However, how each tastant, such as umami, regulates CeA activity, and the correspondence between cells encoding each taste qualities has not been fully elucidated, even under untreated naïve conditions. To investigate innate responses to various tastants in the CeA, we first performed in vivo calcium imaging for two major genetically identified CeA cell populations, *Prkcd*-positive and *Sst*-positive neurons. Mice were sequentially given water and bitter, sweet, and umami tastant solutions as shown in Fig. 2A. Some neurons showed responses prior to presentation of tastant solutions (Additional file 1: Movie S1 and Additional file 2: Movie S2). So we evaluated the difference in responses to water and each tastant solution to minimize the influences of physical stimuli such as oral insertion of a ball tip needle and non-taste-specific responses to drinking itself, and to extract taste-specific response neurons. In *Prkcd*-positive neurons, the largest populations (19.7%) responded to bitter tastant (Fig. 2B), as was reported in previous *Fos*-labeling studies [2, 31]. Notably, a comparative number of neurons (18.8%) also responded to umami, and a smaller number of neurons (7.2%) responded to sweet tastant (Fig. 2B, D, Additional file 3: Fig. S4A, B). Furthermore, we found that 17.8%, 11.0%, and 11.0% of *Sst*-positive neurons responded to umami, bitter, and sweet tastants, respectively (Fig. 2C, Additional file 3: Fig. S5A, B). Interestingly, one-third of sweet-response and one-fifth of umami-response *Sst*-positive neurons also responded to umami and sweet tastants, respectively, both of which are thought to be attractive tastants (Fig. 2C, E, Additional file 3: Fig. S5B). Taken together, both *Prkcd*-positive and *Sst*-positive neurons are not unique populations to respond to a particular tastant, but are composed of mixed cells that respond to negative and positive tastants, although there is a bias in the tendency of the responding tastant.

### Prkcd-positive neurons in the CeA were activated by umami after prolonged umami exposure

*Prkcd*-positive neurons of the CeA were thought to respond to bitter and suppress appetitive behavior [2, 31], but our calcium imaging results indicate that a part of *Prkcd*-positive neurons also respond to attractive taste umami. To elucidate the umami taste information processing in more detail, we investigated the responses of neurons in the CeA and upstream nuclei of the gustatory circuit: the NTS, PB, VTA, and IC. To evaluate the neuronal activities of these nuclei with regard to umami tastant, we performed *Fos* counting studies by fluorescence in situ hybridization (FISH). For the identification of these nuclei, we also used molecular marker genes, including protein kinase *Prkcd* and peptide hormone *Sst* in the CeA, nitric oxide synthase *Nos1* in the IC, tyrosine hydroxylase (*Th*) in the VTA and NTS, and peptide hormones *Calca* and *Adcyap1* in the PB (Additional file 3: Fig. S6A). The *Fos* antisense probe detected *Fos*-positive neurons in the pentylenetetrazole-treated mouse hippocampus, but not in the vehicle-treated mouse hippocampus. Sense probes did not detect the signal in mice hippocampi from both treatment groups (Additional file 3: Fig. S6B).

**Fig. 3:**
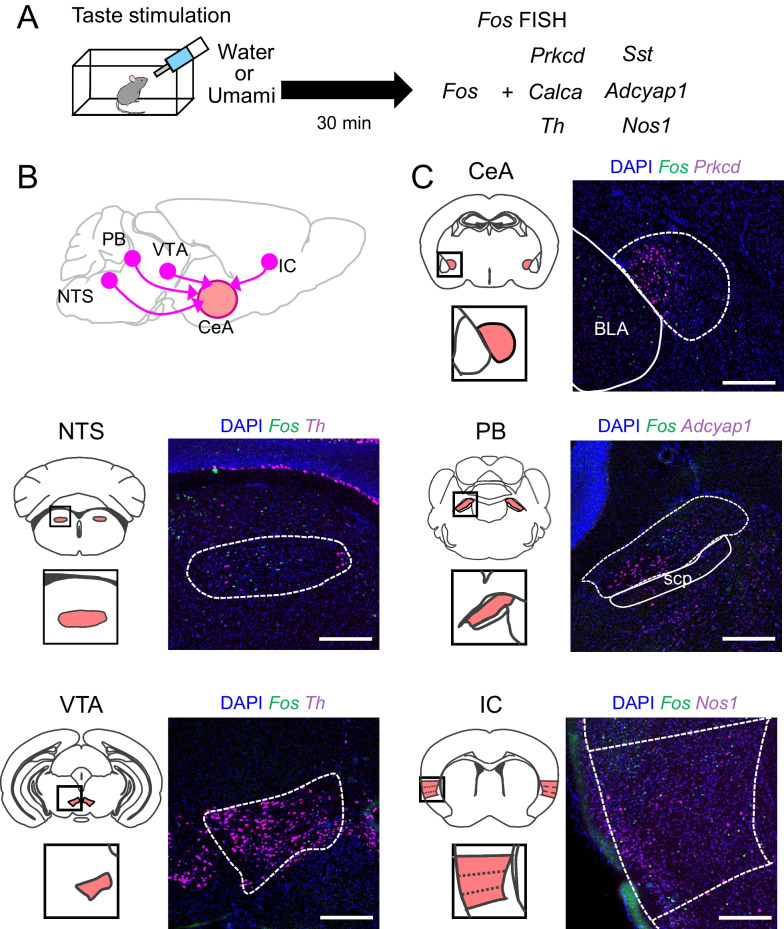
Experimental design of the *Fos* fluorescent in situ hybridization (FISH) assay. **A** Time course of mice brain sampling. **B** Circuit model of afferent projections of the CeA. **C** Representative images of the *Fos* FISH assay. Blue: DAPI, Green: *c-Fos*, Magenta: brain region- or cell type-specific markers. Each scale bar represents 300 μm. Central amygdala (CeA), nucleus tractus solitarius (NTS), lateral parabrachial nucleus (lPB), ventral tegmental area (VTA), insular cortex (IC)

**Fig. 4:**
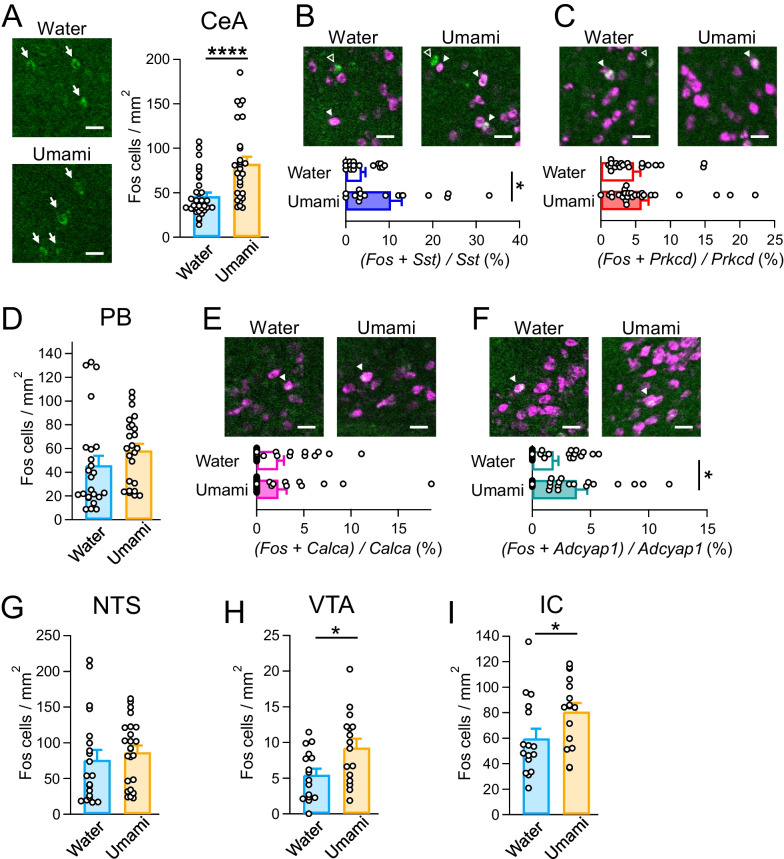
*Fos* FISH assay of single tastant treatment. **A** *Fos* FISH assay at the CeA. (Left) Representative images of the CeA after single water or umami treatment. *Fos*-positive cell counts/1 mm^2^ were not significantly different. Water, n = 32 slices from N = 8 mice; umami, n = 28 from N = 7. **B**, **C** Double *Fos* FISH assay with *Sst* or *Prkcd* markers. The ratios of *Fos*-positive neurons per each marker were not significant. Open and filled triangles indicate single- and double-positive cells, respectively. *Sst*: water, n = 16 from N = 4; umami, n = 16 from N = 4; *Prkcd*: water, n = 20 from N = 5; umami, n = 28 from N = 7. **D**, **G**–**I** *Fos* FISH assay in the PB, NTS, VTA, and IC, which are upstream regions of the CeA. *Fos*-positive cell counts/1 mm^2^ were not significantly different. PB: water, n = 24 from N = 6; umami, n = 24 from N = 6; NTS: water, n = 20 from N = 5; umami, n = 24 from N = 6; VTA: water, n = 16 from N = 4; umami, n = 16 from N = 4; IC: water, n = 16 from N = 4; umami, n = 16 from N = 4. **E**, **F** Double *Fos* FISH assay with *Calca* or *Adcyap1* markers in the PB. The ratios of *Fos*-positive neurons per each marker were not significant. Filled triangles indicate double-positive cells. *Calca*: water, n = 24 from N = 6; umami, n = 24 from N = 6; *Adcyap1*: water, n = 24 from N = 6; umami, n = 24 from N = 6. Each scale bar represents 25 μm. *p < 0.05, ****p < 0.0001 (unpaired *t*-test)

Initially, to investigate the immediate neuronal activity of the tastant, mice were individually housed with restricted feeding for over an hour and restricted drinking for 19–21 h before the taste experiment. To assess the innate taste response, naïve mice were exposed the water, umami, or bitter solutions (Fig. 3). However, the mice provided with bitter solution did not drink it (water, 0.37 ± 1.10 g; umami, 0.58 ± 0.11 g; bitter, 0.03 ± 0.01 g; F_2,22_ = 11.29, *p* = 0.0004, one-way ANOVA; Umami vs. Water group, *p* = 0.2188; Umami vs. Bitter group, *p* = 0.0004; Bitter vs. Water group, *p* = 0.0137, Tukey’s post hoc test). Therefore, we did not perform *Fos* FISH experiments in mice provided with bitter solution (Fig. 4). The *Fos*-positive neurons were increased in the CeA in umami-stimulated mice compared with water-stimulated mice (*p* < 0.0001; Fig. 4A). In addition, we investigated cell type-specific neuronal activity in the CeA by analyzing *Fos* and *Prkcd-* or *Sst*-double-positive neurons. The ratios of *Fos* and *Sst* double-positive neurons per *Sst*-positive neurons in the Umami group was significantly higher than those in the Water group, while *Fos*-positive neurons in the *Prkcd*-positive neurons was comparable between these groups (*Sst*, *p* = 0.016398; *Prkcd*, *p* = 0.4373; Fig. 4B, C). Next, we performed the *Fos* FISH assay in the CeA upstream gustatory nuclei (PB, NTS, VTA, and IC). In the IC, we focused on the area between Bregma + 1.1 mm and + 0.6 mm as the umami field, because the umami field is between the bitter and sweet hot fields [5, 17]. In the PB, we also calculated the ratio of *Fos*-positive neurons in the *Calca*- or *Adcyap1*-positive neurons, because these neurons are known to innervate the CeA [32, 33]. The number of *Fos*-positive neurons in the PB was not significant between the Water and Umami groups (PB, *p* = 0.2181; Fig. 4D). On the other hand, while *Fos*-positive neurons in the *Calca*-positive neurons in the PB was comparable between two groups, *Fos*-positive neurons in the *Adcyap*-positive neurons was increased in the Umami-tastant provided group (*Calca*, *p* = 0.9617; *Adcyap*, *p* = 0.0495; Fig. 4E, F). The NTS showed no difference in *Fos*-positive neurons, but *Fos*-positive neurons in the VTA and IC increased in the Umami group (NTS, *p* = 0.5137; VTA, *p* = 0.0174; IC, *p* = 0.0476; Fig. 4G–I). These results suggest that the nuclei in higher gustatory circuit, such as CeA, VTA, and IC are more activated by the umami administration than NTS and PB, which are the primary nuclei.

**Fig. 5:**
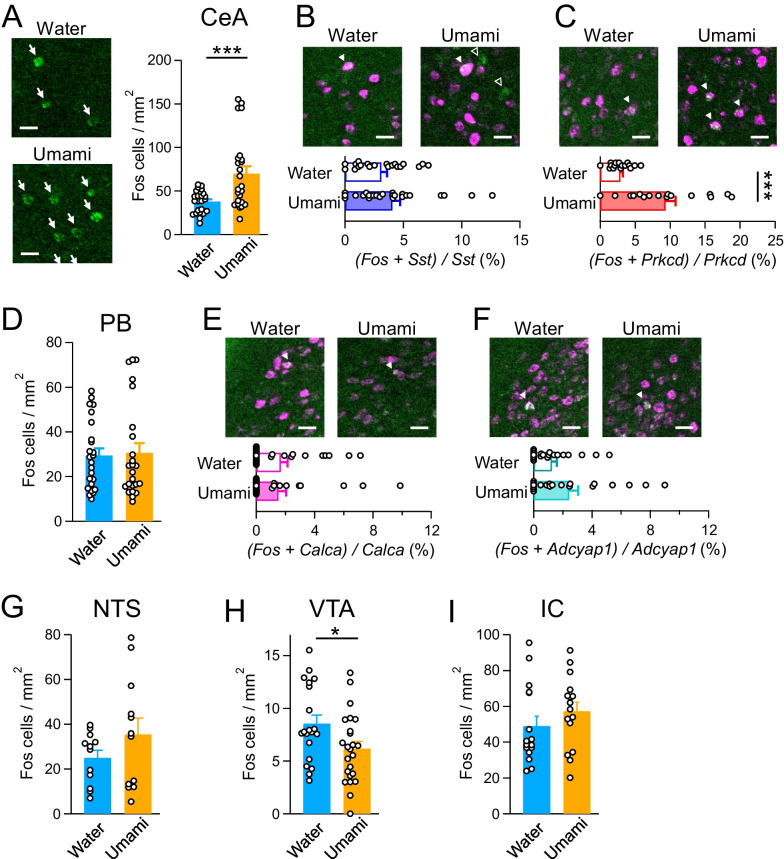
*Fos* FISH assay of prolonged taste exposure mice. **A** *Fos* FISH assay at the CeA. (Left) Representative images of the CeA of the prolonged taste exposure mice after water or umami stimulation. *Fos*-positive cell counts/1 mm^2^ were increased by umami stimulation in the umami-exposed mice. Water, n = 24 slices from N = 6 mice; umami, n = 24 from N = 6. **B**, **C** Double *Fos* FISH assay with *Sst* or *Prkcd* markers. The ratio of *Fos*-positive neurons per each marker was increased in the *Prkcd*-positive neurons. *Sst*: water, n = 20 from N = 5; umami, n = 24 from N = 6; *Prkcd*: water, n = 16 from N = 4; umami, n = 16 from N = 4. **D**, **G**–**I** *Fos* FISH assay in the PB, NTS, VTA, and IC, which are upstream regions of the CeA. *Fos*-positive cell counts/1 mm^2^ were not significantly different. PB: water, n = 24 from N = 6; umami, n = 24 from N = 6; NTS: water, n = 12 from N = 3; umami, n = 12 from N = 3; VTA: water, n = 20 from N = 5; umami, n = 24 from N = 6; IC: water, n = 16 from N = 4; umami, n = 16 from N = 4. **E**, **F** Double *Fos* FISH assay with *Calca* or *Adcyap1* markers in the PB. The ratios of *Fos*-positive neurons per each marker were not significant. *Calca*: water, n = 24 from N = 6; umami, n = 24 from N = 6; *Adcyap1*: water, n = 20 from N = 5; umami, n = 20 from N = 5. Each scale bar represents 25 μm. Arrows indicate *Fos*-positive cells. Open and filled triangles indicate single- and double-positive cells, respectively. *p < 0.05, ***p < 0.001 (unpaired *t*-test)

Next, to determine changes in neuronal activity by prolonged taste exposure, mice received water or umami solution ad libitum for 3 weeks, and *Fos* FISH assay was performed after taste stimulation (Fig. 5). As observed in the single taste stimulation (Fig. 4), *Fos* expression in the CeA was markedly increased by umami stimulation in prolonged taste exposure mice (*p* = 0.0008; Fig. 5A). Interestingly, there was no difference in the ratio of *Fos*-positive neurons in the *Sst*-positive neurons, while the ratio of *Fos*-positive neurons in the *Prkcd*-positive neurons was significantly increased in the Umami group (*Prkcd*, *p* = 0.0001; *Sst*, *p* = 0.2778; Fig. 5B, C). Among the higher gustatory nuclei, no difference was observed except for the VTA, unlike the single taste administration (PB, *p* = 0.8423; *Calca*, *p* = 0.8279, *Adcyap1*, *p* = 0.1059; NTS, *p* = 0.2047; IC, *p* = 0.2740; Fig. 5D–G, H). Intriguingly, the VTA showed a decrease in the *Fos*-positive neurons in the prolonged umami administration (VTA, *p* = 0.0276; Fig. 5H). These results suggest that prolonged exposure to umami taste induces some plastic changes in the gustatory circuit, particularly in the CeA, in a cell type-specific manner.

## Discussion

The modification of taste preference by previous taste experiences has been studied in animals. In rodents, both attractive and aversive taste exposure increases intake of the exposed taste; exposure to umami in adulthood or sweet in the lactation period enhances its palatability [14, 34], and exposure to bitter in post-weaning or adulthood, or sour in the lactation period reduces its aversiveness [10, 12]. Our results are consistent with this body of evidence and showed that prolonged exposure to umami and bitter in the post-weaning juvenile period also increases the preference for the exposed taste. Furthermore, we found that prolonged exposure to umami did not affect bitter preference, and vice versa for bitter exposure, which suggests that there is little crossover effect of different taste qualities. In contrast to our results, it has also been reported that bitter exposure during lactation has no significant effect on bitter ingestion [10]. Furthermore, sweet exposure in the post-weaning period reduces its hedonic valence in adulthood [13]. These lines of evidence suggest that appropriate time window of taste experiences may be critical for the increment of taste preference. It would be an interesting future study to examine whether there is a critical period to induce such experience-dependent changes in taste preference. The studies described above mainly focused on preference for the exposed taste, but not other tastes, especially opposite valence tastes such as umami and bitter. We therefore examined the influence on bitter and umami preference in the Umami and Bitter groups and showed that preference for the unexposed taste is unchanged, which suggests that taste preference is modified in a manner selective to the exposed taste.

We used MSG- and MPG-based umami solutions for the two-bottle tests. The Water group did not show a strong umami preference using MSG-based umami (Fig. 1C, E), while it did using MPG-based umami (Additional file 3: Fig. S2C, E). The reason for this is unclear. One can speculate that there may be some interaction between sodium and umami signals. For example, a previous study demonstrated that some Satb2-positive neurons in PB respond to both sodium chloride and umami stimuli [16]. These Satb2 neurons enhance taste perception and affect licking behavior. One possibility is that the umami solution containing 100 mM MSG influences taste perception and umami intake. Therefore, the slight difference in preference between MSG- and MPG-based umami in the Water group may be due to the difference in the neuronal activities in the PB to umami and sodium signals. The interaction and plasticity mechanisms are essential topics for future investigation.

In the present study, we first targeted and investigated the CeA neurons in response to various tastants because these neurons receive direct and indirect inputs from multiple nuclei of the gustatory circuit, and play a critical role in encoding negative or positive valence. The taste response of the CeA neurons has been investigated in rodents by several previous *Fos*-labeling studies; however, it should be noted that each experiment employed a partially different method of taste stimulation. Otsubo et al. reported that the *Fos*-like immunoreactivity of neurons in the CeA was increased by both forcibly sweet and umami stimulations after 24-h fasting compared with salty stimulation [35]. In contrast, Cai et al. reported that *Fos*-positive neurons in the CeA were increased by forcible intraoral infusion of bitter tastant solution but not sweet tastant solution [2]. Furthermore, Kim et al. employed free access to bitter tastant solution after 24 h of water deprivation and found that *Fos*-positive neurons were increased specifically in *Prkcd*-positive neurons in the capsular part of the CeA compared with mice provided with neutral-taste water [31]. Collectively, the taste specificity of the CeA cell-types remains to be ambiguous. Therefore, in order to clarify not only the responsiveness to various tastants but also the correspondency of responding cells, here we attempted to consecutive recording of neuronal responses to various tastants using calcium imaging, and identified that the *Prkcd*- and *Sst*-positive neuronal populations consisted of both cells responding to negative and positive tastants, respectively. The CeA receives direct inputs from bitter-responsive neurons in the PB and bitter-responsive neuron hotspots located in the caudal part of the IC [2, 5], suggesting that the bitter-responsive neurons of the CeA can be activated by these inputs. Since the IC also possesses a sweet-responsive neuron hotspot on the rostral side and mainly projects to the basolateral amygdala (BLA), it is possible that sweet stimulation, at least in part, is indirectly transmitted to the CeA through the BLA [5, 17]. In addition, it has been reported that an umami-responsive neuron hotspot also exists in the IC between the bitter- and sweet-responsive neuron hotspots [17], and there are direct inputs to the CeA from these areas of the IC [36], suggesting that umami information can be transmitted to the CeA directly from the umami hotspot of the IC. Furthermore, the VTA dopamine neurons projects directly to the CeA, especially to the medial part of the CeA (CeM), where *Prkcd*-positive neurons are less abundant and *Sst*-positive neurons are more abundant [15, 37]. Therefore, it is also possible that umami information is relayed to *Sst*-positive neurons in the CeA via VTA dopamine neurons. Interestingly, some dopaminergic neurons in the VTA project to the IC, consolidating aversive taste memory [38]. In order to elucidate through which nuclei the information for each taste is relayed to the CeA, further studies in combination with circuit tracing are required. Together, our findings suggest that taste stimuli are represented in a more complex manner in the CeA than previously thought.

It is noteworthy that the ratio of the *Fos*-positive neurons in the CeA were high in the *Sst*-positive neurons in single umami administration, while they were predominant in the *Prkcd*-positive neurons in prolonged umami administration. At least, our calcium imaging showed that there is a population that responds to umami in *Prkcd*-positive neurons, suggesting that prolonged umami administration enhanced the activity of these neurons as well. Because the *Prkcd*-positive and *Prkcd*-negative (mainly *Sst*-positive) neurons are both inhibitory neurons and form reciprocally connected microcircuits in the CeA [39, 40], one possible underlying mechanism is that *Sst*- and *Prkcd*-positive neurons are plastically regulated in a different manner via mutual inhibition, resulting in opposite plastic changes. In fact, *Sst*- and *Prkcd*-positive neurons exhibit contradictory responses in fear learning and pain-like behavior [41, 42]. Furthermore, among *Sst*-positive neurons, different subregions within CeA have different plasticity phenotypes [43]. Another possibility is that the inputs to the *Sst*- and *Prkcd*-positive neurons are different, thereby acute and chronic taste experiences have different effects on these cell-types. Indeed, excitatory synaptic inputs from the IC to the lateral and capsular part of the CeA are greater in *Sst*-positive neurons [44], whereas PB inputs are larger in *Sst*-negative neurons in the capsular part of the CeA, but those are larger in *Sst*-positive neurons in the CeM [43]. Also, the VTA dopaminergic neurons project predominantly to the CeM, where *Sst*-positive neurons are rich. Therefore, prolonged umami administration may cause experiment-related plasticity in the CeA to act on *Sst*- and *Prkcd*-positive neurons differentially, resulting in changes in the balance between these neurons which may influence the palatability of umami. To support this notion, it is known that the satiety-related peptide hormone cholecystokinin (CCK) is released by umami [45]. CCK from the peripheral tissue can activate *Prkcd*-positive neurons in the CeA [46]. These lines of evidence suggest that umami experience is involved in activation of the CeA *Prkcd*-positive neurons via the CCK pathway. Although the causal relationship between drinking behavior and activity of *Prkcd*-positive neurons is not clear due to the limitations of our experimental methods, these neurons may intricately regulate umami preference and drinking control. Intriguingly, a previous study reported that the CeA *Prkcd*-positive neurons are critically involved in chronic alcohol-drinking behavior in rats [47]. Therefore, one possibility is that the *Prkcd*-positive neurons are involved in experience-dependent plastic changes such as prolonged umami intake and chronic alcohol drinking. It would be an interesting future study to examine the molecular mechanisms of the synaptic plasticity in the *Prkcd*-positive neurons and their physiological consequences.

Although the present study focused on the plasticity to the central nervous system caused by taste experiences, it is also possible that changes in the periphery influence taste preference. In general, animals avoid bitter tastes, but frequent ingestion decreases the avoidance behavior. The decrease in aversiveness may be due to fewer bitter taste receptors. The bitter receptor of quinine, the bitter substance used in the present study, has been identified [48], and it is possible that the sensitivity of some of these receptors was changed. Peripheral nerves have been reported to sense bitterness and nutrition and contribute to preference [49]. In addition, it has also been suggested that changes in peripheral taste bud structure are accompanied by changes in preference due to taste experience [34]. In the present study, we were unable to examine the peripheral involvement in changes in taste preference, but this will need to be examined in the future.

Experimental systems such as conditioned taste aversion and conditioned place aversion exist for the mechanism that makes animals dislike what they like, and these systems have been widely studied worldwide [2, 3, 50, 51]. Conversely, there are few experimental systems that examine the mechanism of liking something one dislikes, and further research on the mechanism of increased preference by bitter taste experience may lead to the elucidation of a complementary mechanism. Unfortunately, in the present study, *Fos* FISH analysis was not possible for bitter taste in the free-moving condition. In future studies, we would like to examine changes in *Fos* expression patterns associated with single and prolonged changes in bitter taste exposure by either lowering the concentration of bitter taste or by forced drinking. Furthermore, we would like to research changes in umami preference behavior through the inhibition of *Prkcd*-positive neurons by the artificial circuit manipulation of neuronal activity during prolonged umami exposure.

## Supplementary Information

**Additional file 1: Movie S1.** Representative calcium imaging movie of *Prkcd-*cre mice (right) and simultaneously recorded behavioral movie (left). The tastant solution was poured at the timing when the text color is reversed. The movie plays at 2× speed.

**Additional file 2: Movie S2.** Representative calcium imaging movie of *Sst-*cre mice (right) and simultaneously recorded behavioral movie (left). The tastant solution was poured at the timing when the text color is reversed. The movie plays at 2× speed.

**Additional file 3: Figure S1.** Time course of access duration in two-bottle test. **A**–**C** Access duration to water or umami bottles every 5 min in 2 days two-bottle tests in Water (A), Umami (B) and Bitter (C) groups. **D**–**F** Access duration to water or bitter bottles every 5 min in 2 days two-bottle tests in Water (D), Umami (E) and Bitter (F) groups. Data are represented as mean ± SEM. Water group, n = 8; Umami group, n = 9; Bitter group, n = 7. *p < 0.05, **p < 0.01, ***p < 0.001 (Paired *t*-test). **Figure S2.** Preference for MPG-based umami in the two-bottle test in prolonged taste exposure mice. **A** Experimental paradigm of prolonged taste exposure and two-bottle test. **B** Intake of water and umami during 15-min two-bottle test. **C** Preference ratios of umami. Preference ratios were calculated as the ratio of the umami intake to the total intake. **D** Access duration to water or umami bottle. **E** Access ratio of umami bottle. Each circle represents results from one mouse. Data are represented as mean ± SEM. Water group, n = 10; MPG group, n = 10. *p < 0.05, **p < 0.01 (paired t-test); ^†^*p* < 0.05, ^††^*p* < 0.01 (one sample t-test); ^$^*p* < 0.05 (Welch’s t-test followed by correction with Bonferroni method). **Figure S3.** **A** Schematic of viral injections and lens implantation into the CeA for calcium imaging. **B** Representative image of GCaMP6f expression and lens probe tract of *Prkcd-*cre mouse brain. Scale bar, 200 µm. **C** Implanted lens probe locations of four *Prkcd-*cre (magenta) and four *Sst-*cre (light blue) mice. The values indicate anterior–posterior distances from bregma. **Figure S4.** Heatmaps indicate responses to umami (upper), bitter (middle), and sweet (lower) tastant solution of 3 trials each (A), and average responses of 3 trials for each tastant (B) in total extracted cell population from *Prkcd*-cre mice (223 cells) aligned in descending order by response value for umami (upper), bitter (middle), and sweet (lower) described in the methods section. Red lines on the left of each row correspond to neurons activated in each taste. **Figure S5.** Heatmaps indicate responses to umami (upper), bitter (middle), and sweet (lower) tastant solution of 3 trials each (A), and 3-trial average responses for each tastant (B) in total extracted cell population from *Sst*-cre mice (191 cells) aligned in descending order by response value for umami (upper), bitter (middle), and sweet (lower) described in the methods section. Red lines on the left of each row correspond to neurons activated in each taste. **Figure S6.** Validation of FISH probes. (A) Validation of probes for the brain region or cell type-specific markers *Prkcd*, *Sst*, *Nos1*, *Th*, *Calca*, and *Adcyap1*. Brain region or cell type-specific signals were observed by antisense (AS) probes, but not by sense (S) probes. (B) Validation of the *Fos* probe. Saline (control) or Pentylenetetrazol (Ptz) treated mice were used for the *Fos* FISH assay with *Fos* AS or S probes. *Fos*-positive signals at the hippocampal dentate gyrus were observed in the Ptz-treated and AS probe groups.

## Data Availability

All data are available upon request to the corresponding author.

## Abbreviations

CeA: Central amygdala
NTS: Nucleus of the solitary tract
PB: Parabrachial nucleus
VTA: Ventral tegmental area
IC: Insular cortex
AAV: Adeno-associated virus
GRIN: Gradient index
cRNA: Complementary RNA
FISH: Fluorescence in situ hybridization
NOS1: Nitric oxide synthase 1
Prkcd: Protein kinase C delt
Sst: Asomatostatin
Th: Tyrosine hydroxylase
Calca: Calcitonin gene-related peptide
Adcyap1: Pituitary adenylate cyclase-activating polypeptide
FITC: Fluorescein isothiocyanate
DIG: Digoxigenin
SSC: Standard saline citrate
DAPI: 4′,6-Diamidino-2-phenylindole
BLA: Basolateral amygdala
CeM: Medial part of the CeA
MSG: Monosodium glutamate
MPG: Monopotassium glutamate
CCK: Cholecystokinin
