# Supplementary material for: Experience-dependent changes in affective valence of taste in male mice
Source: Mol Brain. 2023 Mar 11;16:28. doi: 10.1186/s13041-023-01017-x (PMC10007816; doi:10.1186/s13041-023-01017-x)
Supplement: Supplementary file 3 — Additional file 3: Figure S1. Time course of access duration in two-bottle test. A–C Access duration to water or umami bottles every 5 min in 2 days two-bottle tests in Water (A), Umami (B) and Bitter (C) groups. D–F Access duration to water or bitter bottles every 5 min in 2 days two-bottle tests in Water (D), Umami (E) and Bitter (F) groups. Data are represented as mean ± SEM. Water group, n = 8; Umami group, n = 9; Bitter group, n = 7. *p < 0.05, **p < 0.01, ***p < 0.001 (Paired t-test). Figure S2. Preference for MPG-based umami in the two-bottle test in prolonged taste exposure mice. A Experimental paradigm of prolonged taste exposure and two-bottle test. B Intake of water and umami during 15-min two-bottle test. C Preference ratios of umami. Preference ratios were calculated as the ratio of the umami intake to the total intake. D Access duration to water or umami bottle. E Access ratio of umami bottle. Each circle represents results from one mouse. Data are represented as mean ± SEM. Water group, n = 10; MPG group, n = 10. *p < 0.05, **p < 0.01 (paired t-test); †p < 0.05, ††p < 0.01 (one sample t-test); $p < 0.05 (Welch’s t-test followed by correction with Bonferroni method). Figure S3. A Schematic of viral injections and lens implantation into the CeA for calcium imaging. B Representative image of GCaMP6f expression and lens probe tract of Prkcd-cre mouse brain. Scale bar, 200 µm. C Implanted lens probe locations of four Prkcd-cre (magenta) and four Sst-cre (light blue) mice. The values indicate anterior–posterior distances from bregma. Figure S4. Heatmaps indicate responses to umami (upper), bitter (middle), and sweet (lower) tastant solution of 3 trials each (A), and average responses of 3 trials for each tastant (B) in total extracted cell population from Prkcd-cre mice (223 cells) aligned in descending order by response value for umami (upper), bitter (middle), and sweet (lower) described in the methods section. Red lines on the left of each row correspond [file 13041_2023_1017_MOESM3_ESM.pdf]

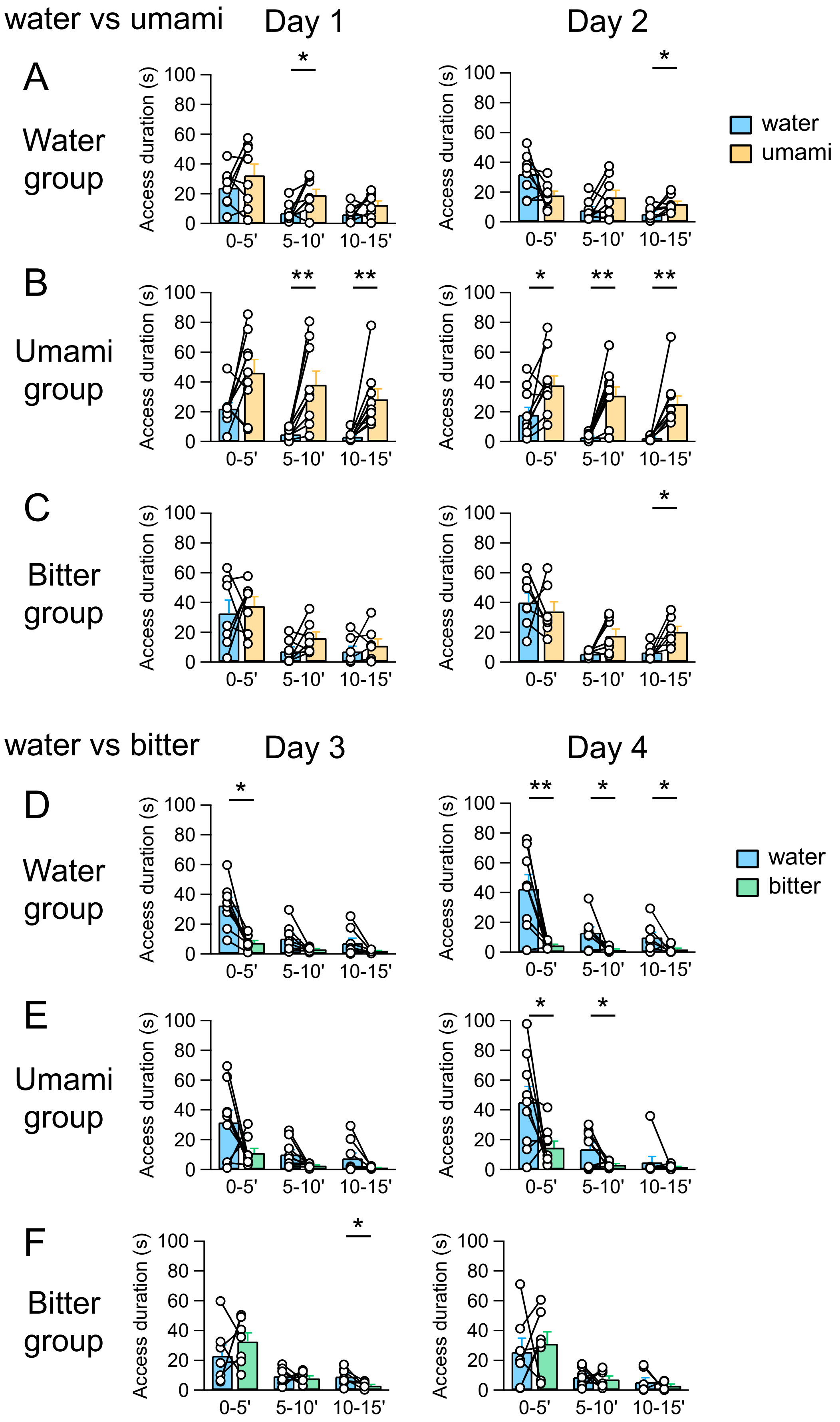

Figure S1. Time course of access duration in two-bottle test.
(A-C) Access duration to water or umami bottles every 5 min in two days two-bottle tests in Water (A), Umami (B) and Bitter (C) groups. **(D-F)** Access duration to water or bitter bottles every 5 min in two days two-bottle tests in Water (D), Umami (E) and Bitter (F) groups. Data are represented as mean ± SEM. Water group, n = 8; Umami group, n = 9; Bitter group, n = 7. *$p < 0.05$, **$p < 0.01$, ***$p < 0.001$ (Paired *t*-test).

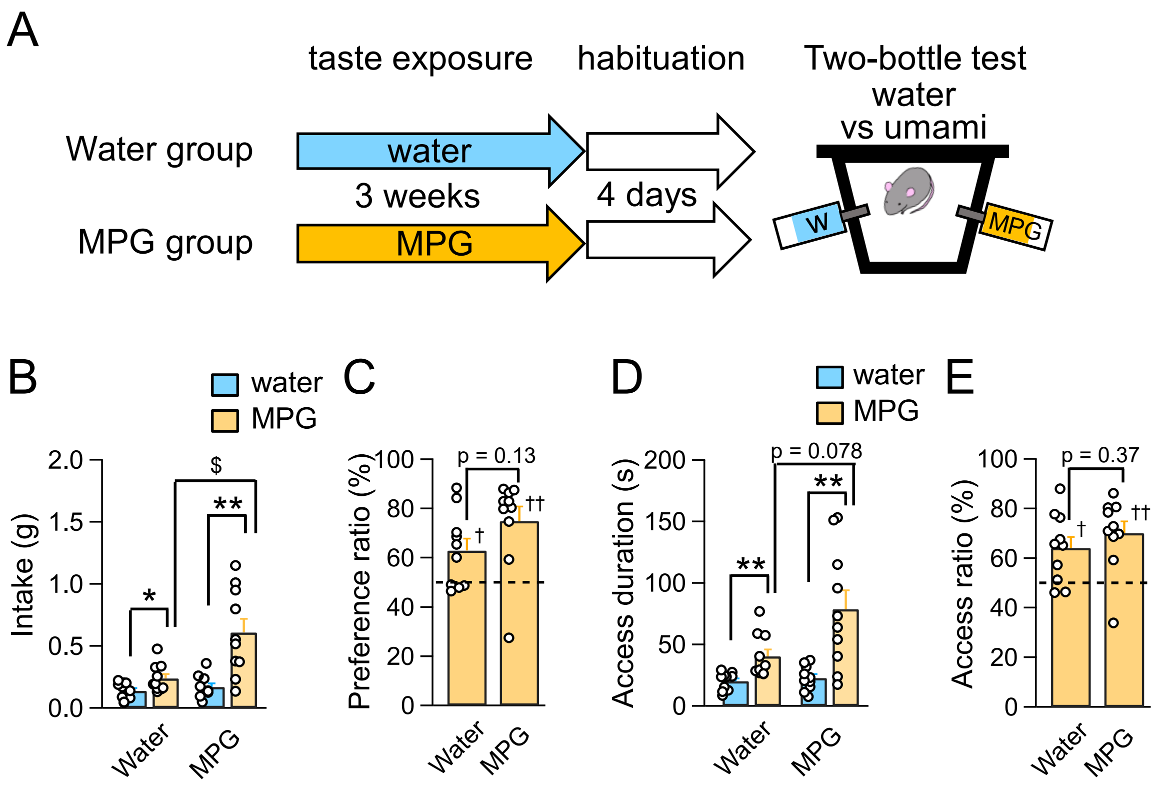

Figure S2. Preference for MPG-based umami in the two-bottle test in prolonged taste exposure mice.
(A) Experimental paradigm of prolonged taste exposure and two-bottle test. **(B)** Intake of water and umami during 15-min two-bottle test. **(C)** Preference ratios of umami. Preference ratios were calculated as the ratio of the umami intake to the total intake. **(D)** Access duration to water or umami bottle. **(E)** Access ratio of umami bottle. Each circle represents results from one mouse. Data are represented as mean ± SEM. Water group, n = 10; MPG group, n = 10. *p < 0.05, **p < 0.01 (paired t-test); †p < 0.05, ††p < 0.01 (one sample t-test); $^{\$}p$ < 0.05 (Welch's t-test followed by correction with Bonferroni method).

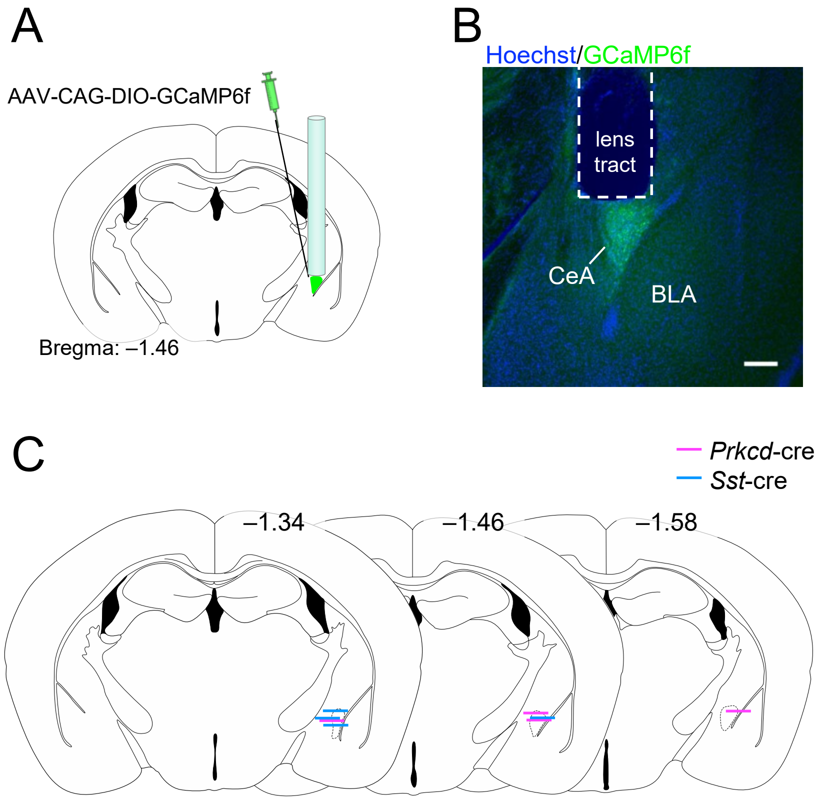

Figure S3.
(A) Schematic of viral injections and lens implantation into the CeA for calcium imaging. **(B)** Representative image of GCaMP6f expression and lens probe tract of *Prkcd*-cre mouse brain. Scale bar, 200 µm. **(C)** Implanted lens probe locations of four *Prkcd*-cre (magenta) and four *Sst*-cre (light blue) mice. The values indicate anterior–posterior distances from bregma.

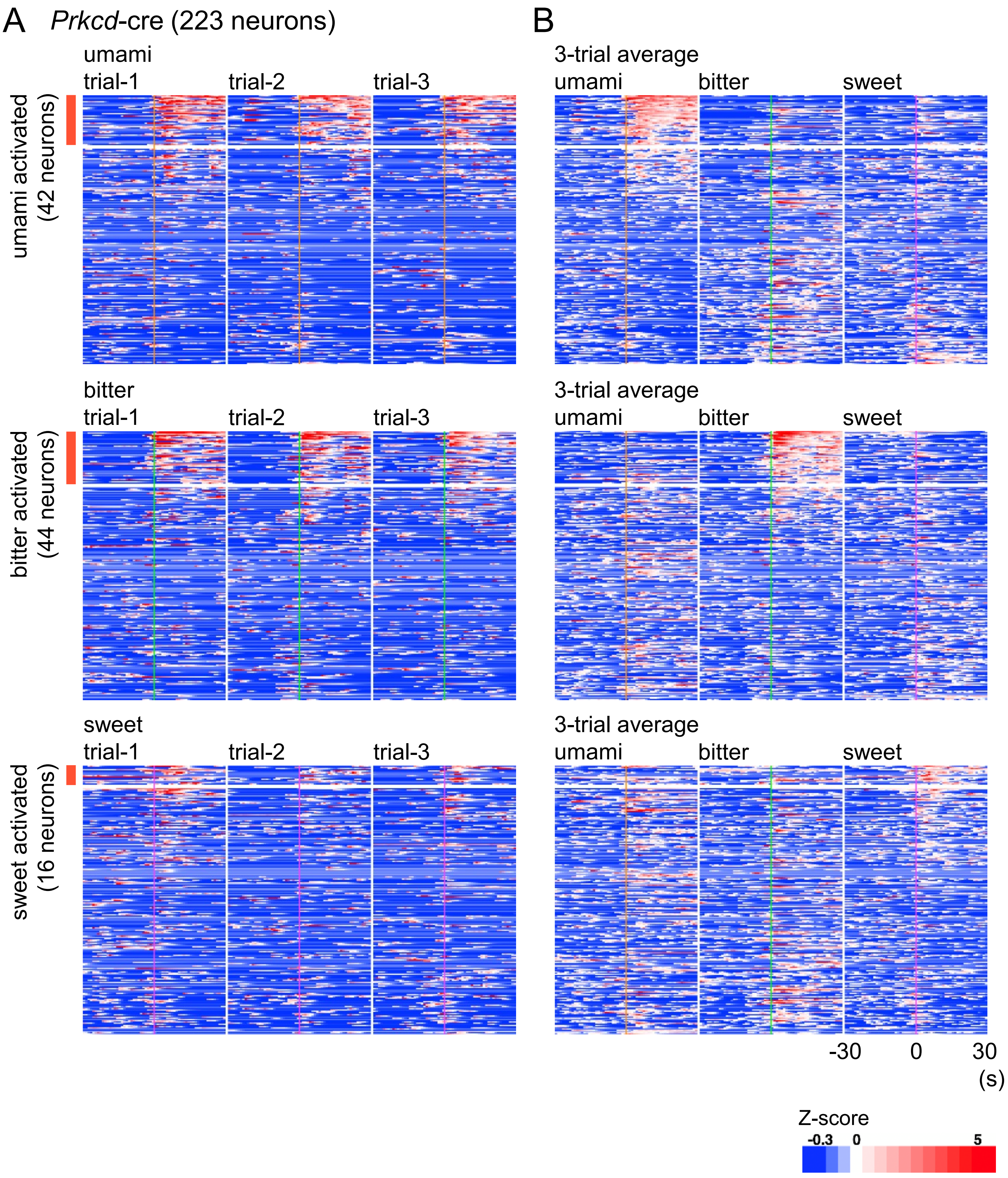

Figure S4.

Heatmaps indicate responses to umami (upper), bitter (middle), and sweet (lower) tastant solution of 3 trials each (A), and average responses of 3 trials for each tastant (B) in total extracted cell population from *Prkcd*-cre mice (223 cells) aligned in descending order by response value for umami (upper), bitter (middle), and sweet (lower) described in the methods section. Red lines on the left of each row correspond to neurons activated in each taste.

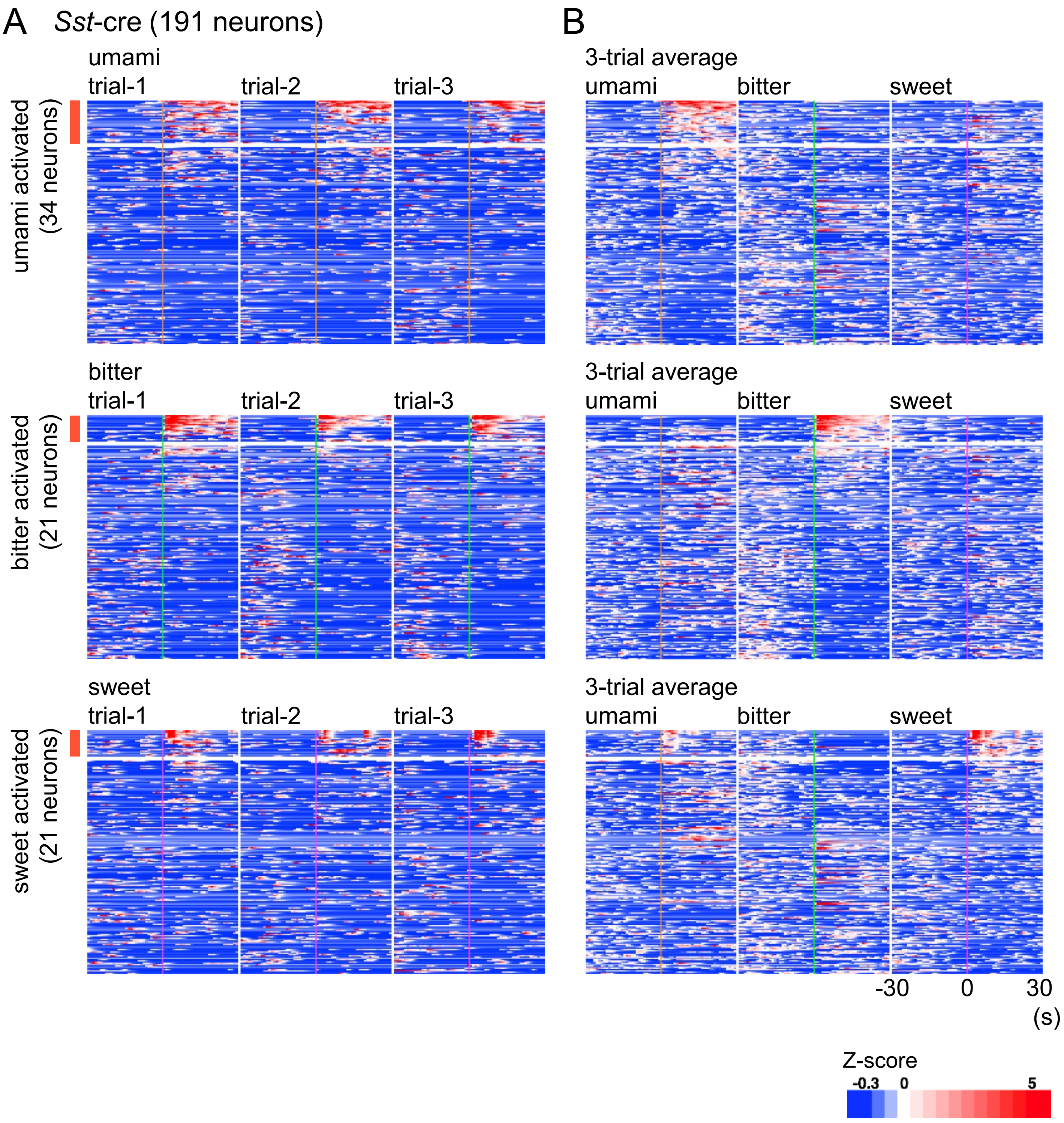

Figure S5.
Heatmaps indicate responses to umami (upper), bitter (middle), and sweet (lower) tastant solution of 3 trials each (A), and 3-trial average responses for each tastant (B) in total extracted cell population from *Sst*-cre mice (191 cells) aligned in descending order by response value for umami (upper), bitter (middle), and sweet (lower) described in the methods section. Red lines on the left of each row correspond to neurons activated in each taste.

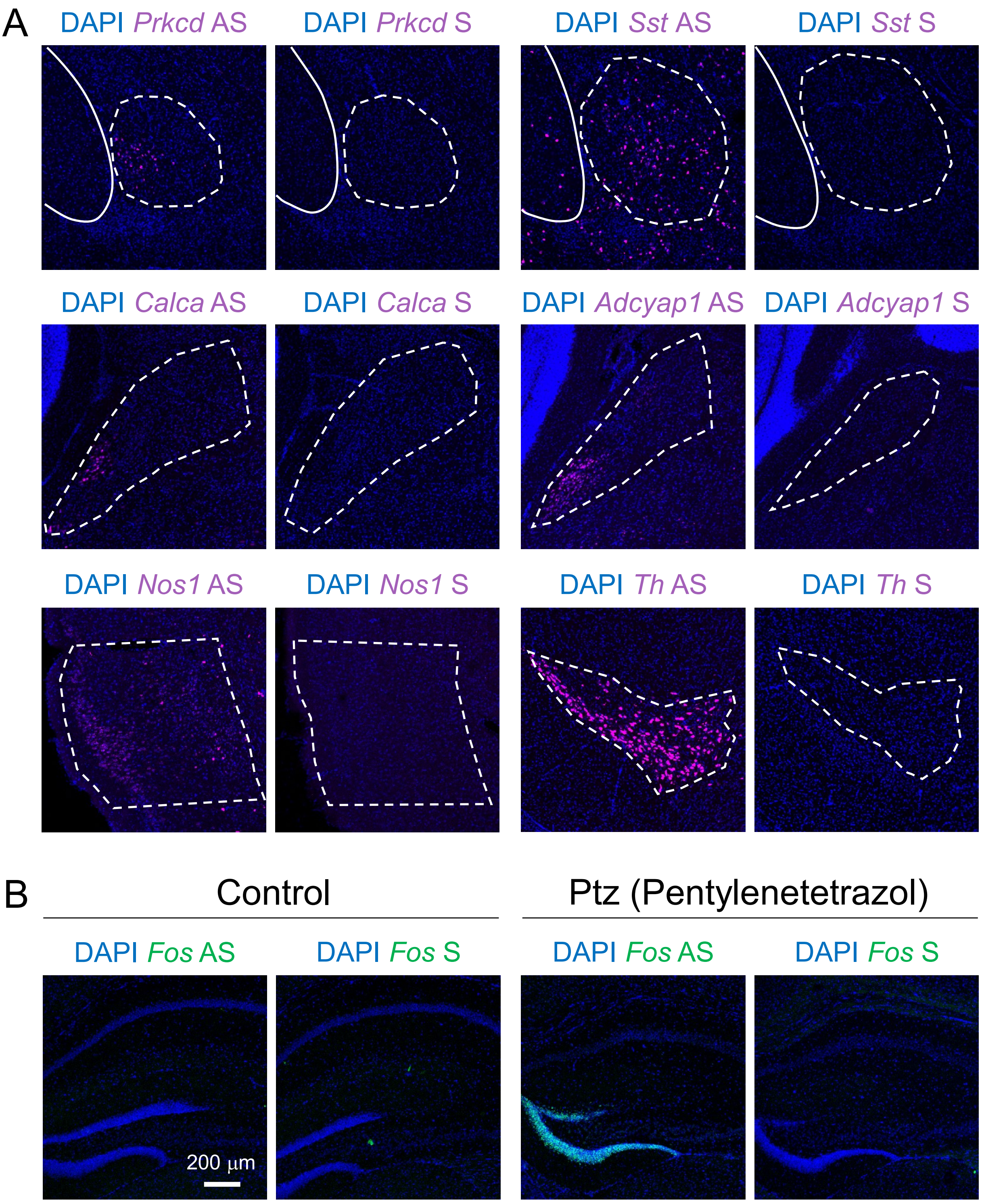

Figure S6. Validation of FISH probes.
(A) Validation of probes for the brain region or cell type-specific markers *Prkcd*, *Sst*, *Nos1*, *Th*, *Calca*, and *Adcyap1*. Brain region or cell type-specific signals were observed by antisense (AS) probes, but not by sense (S) probes. **(B)** Validation of the *Fos* probe. Saline (control) or Pentylenetetrazol (Ptz) treated mice were used for the *Fos* FISH assay with *Fos* AS or S probes. *Fos*-positive signals at the hippocampal dentate gyrus were observed in the Ptz-treated and AS probe groups.
